# Effect of aerobic exercise intensity on health-related quality of life in severe obesity: a randomized controlled trial

**DOI:** 10.1186/s12955-022-01940-y

**Published:** 2022-02-24

**Authors:** Jarle Berge, Jøran Hjelmesæth, Ronette L. Kolotkin, Øyvind Støren, Solfrid Bratland-Sanda, Jens Kristoffer Hertel, Espen Gjevestad, Milada Cvancarova Småstuen, Jan Helgerud, Tomm Bernklev

**Affiliations:** 1 Morbid Obesity Centre, Vestfold Hospital Trust, Box 2168, 3103 Tønsberg, Norway; 2 Clinic of Medicine and Rehabilitation, Vestfold Hospital Trust, Stavern, Norway; 3 Nature, Health and Environment, University of South-Eastern Norway, Bø, Norway; 4 Department of Endocrinology, Morbid Obesity and Preventive Medicine, Institute of Clinical Medicine, University of Oslo, Oslo, Norway; 5 Quality of Life Consulting, Durham, NC USA; 6 Department of Family Medicine and Community Health, Duke University Medical Center, Durham, NC USA; 7 Førde Hospital Trust, Førde, Norway; 8 Department of Sport, Physical Education and Outdoor Life Studies, University of South-Eastern Norway, Bø, Norway; 9 Norwegian Police University College, Stavern, Norway; 10 Department of Circulation and Medical Imaging, Faculty of Medicine and Health Science, Norwegian University of Science and Technology, Trondheim, Norway; 11 Myworkout, Medical Rehabilitation Clinic, Trondheim, Norway; 12 Faculty of Medicine, Institute for Clinical Medicine, University of Oslo, Oslo, Norway; 13 R&D Department, Vestfold Hospital Trust, Tønsberg, Norway

**Keywords:** VO_2max_, Health-related quality of life, Severe obesity, Aerobic exercise

## Abstract

**Background:**

Aerobic exercise is an important part of obesity treatment and may improve health-related quality of life (HRQOL). The objective of this study was to compare the effect of two different exercise programs on health-related quality of life in patients with severe obesity.

**Methods:**

This was a single-center, open-label, randomized, parallel-group study comparing the effects of a 24-week moderate-intensity continuous training (MICT) program and a combined high-intensity interval training program with MICT (HIIT/MICT). The primary objective (specified secondary outcome) was to assess HRQOL by using the general health dimension of the Short Form Health Survey (SF-36). The secondary objectives were to assess other dimensional SF-36 scores, the impact of weight on the physical and psychosocial aspects of quality of life (IWQOL-Lite), and the burden of obesity-specific weight symptoms (WRSM).

**Results:**

73 patients were enrolled and reported patient reported outcome measures, with 71 patients (55% females) allocated to either MICT (n = 34) or HIIT/MICT (n = 37). In the intention-to-treat analysis, general health scores increased between baseline and 24-week follow-up in both the HIIT/MICT group and the MICT group, with a mean change of 13 (95% CI 6–21) points and 11 (95% CI 5–17) points, respectively, with no difference between the groups. The effect sizes of these changes were moderate. The vitality and social functioning scores of SF-36, and the physical function and self-esteem scores of IWQOL-Lite increased moderately in both groups, with no difference between groups. The tiredness, back pain, and physical stamina scores based on WRSM showed moderate to strong changes in both the groups.

**Conclusions:**

Patients who had completed a combined HIIT/MICT program did not experience larger improvements in general health compared with those completing a clean 24-week MICT program. Exercise may confer general health benefits independent of intensity.

***Trials registration*:**

Regional Committees for Medical and Health Research Ethics south east, Norway, October 23, 2013 (identifier: 2013/1849) and ClinicalTrials.gov December 8, 2014 (identifier: NCT02311738).

**Supplementary Information:**

The online version contains supplementary material available at 10.1186/s12955-022-01940-y.

## Introduction

Aerobic exercise contstitutes an important part of obesity treatment and may also improve health-related quality of life (HRQOL) [1, 2], i.e. how well an individual functions in daily life and their perceived well-being [3]. Exercise interventions have documented modest improvements in HRQOL in patients with chronic illness and adolescents with overweight and obesity [4, 5], with more frequent exercise seemingly to be associated with larger improvements in HRQOL [6]. The explanation for this is unclear, but it may be related to increased maximal cardiorespiratory fitness [4, 7–12], as maximal cardiorespiratory fitness is generally low in patients with severe obesity [10]. Aerobic exercise with either high-intensity interval training (HIIT), or moderate-intensity continuous training (MICT), is associated with increased maximal cardiorespiratory fitness, both in persons with normal-weight and obesity [13–16]. However, previous studies have reported larger improvements in maximal cardiorespiratory fitness after HIIT than after MICT [13, 17, 18]. Further, 16 weeks of high-intensity or moderate-intensity exercise that leads to small weight losses was shown to improve general health and physical functioning as measured by the generic HRQOL questionnaire Short Form Health Survey (SF-36) in healthy inactive individuals with a wide range of body mass index (BMI) (33.3–64.8 kg*m^−2^) [19]. In addition, vitality and mental health dimensions improved in the high-intensity group [19]. However, the effects of different aerobic exercise programs on HRQOL are uncertain.

To our knowledge, now previously randomized studies have compared patient reported HRQOL as an outcome in patients with severe obesity participating in a long-term MICT program with the combined HIIT/MICT program, increasing maximal cardiorespiratory fitness.

In light of this, the objective of the present study among treatment seeking patients with severe obesity was to compare the effects of two different exercise interventions on generic HRQOL, with the general health dimension in the SF-36 as the main efficacy variable. This was a pre-specified secondary outcome in a study comparing the effects of these two intervention on 24-week change in energy expenditure during exercise (primary outcome) [20]. We hypothesized that patients completing a combined HIIT/MICT program would experience larger improvements in general health compared with those completing a 24-week MICT program.

## Methods

### Study design and location

This is a single-center, open-label, randomized (1:1), parallel-group study (clinicaltials.gov identifier: NCT02311738) conducted at a tertiary care center at Vestfold Hospital Trust in Tønsberg, Norway. The primary outcome, the 24-week change in energy expenditure during exercise, has been published [20]. Health-related quality of life was a prespecified secondary outcome.

### Patients

Eligible adult patients (≥ 18 years) were those with severe obesity defined as body mass index, BMI ≥ 40.0 kg*m^−2^, or 35.0–39.9 kg*m^−2^ in combination with at least one obesity related co-morbidity and with a stable body weight during the last 3 months (± 5 kg). Exclusion criteria were ascertained by qualified health personnel and included uncompensated heart failure, recent myocardial infarction or stroke during the last 6 months, severe arrhythmia or heart failure, unstable angina pectoris, renal failure, pregnancy, severe eating disorders, active substance abuse, being on a standardized diet, taking medications known to significantly affect appetite or metabolism, and physical immobility. Written informed consent was obtained from all patients. All patients referred to the outpatient clinic were consecutively prescreened with regarding inclusion and exclusion criteria. Those eligible for inclusion were contacted and informed about the study by telephone with those willing to participate undergoing a second medical screening at the center. The study period was from January 5, 2015 to June 9, 2017. The study was approved by the Regional Committees for Medical and Health Research Ethics South East, Norway (2013/1849).

### Exercise interventions

**Fig. 1:**
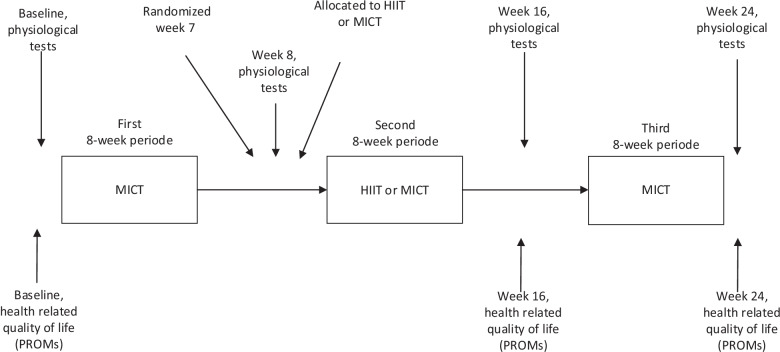
Timeline for visit, PROMs and training. PROMs reported at baseline, 16-week and 24-week

In phase I, to gradually prepare patients for increasing physical activity and to prevent injuries, all patients initially underwent 8-week MICT. Second, those completing phase I were randomized and allocated (1:1 ratio) to either 8-week HIIT or 8-week MICT (Phase II). In phase III, both groups subsequently underwent 8-week MICT (Fig. 1), making it possible to investigate any legacy effects of the mid-term HIIT. The 24 weeks intervention is based on these three exercise phases. Patients were instructed to maintain their habitual dietary intakes during the intervention, with no specific focus on weight loss.

The MICT consisted of a 10-min warm up at 50% of heart rate maximum (HR_max_) (± 3 beats per minutes; BPM), a 35-min exercise at 70% of HR_max_ (± 3 BPM), and a 4-min cool down at 50% of HR_max_ (± 3 BPM). The HIIT consisted of a 10-min warm up at 70% of HR_max_ (± 3 BPM), then 4*4 min at 90–95% of HR_max_, divided by 3-min active recovery periods at 70% of HR_max_ (± 3 BPM), and finally 5 min cool down at 70% of HR_max_ (± 3 BPM). The total time for each of the exercise durations was 49 min for MICT and 40 min for HIIT. Three workouts per week were performed, either cycling or walking/running.

### Outcomes

The main outcome of the present study was to compare the effect of two different 24-week exercise programs on the general health dimension of SF-36 [21, 22]. Secondary outcomes were to compare changes between the two groups in the other dimensions of SF-36 scores; physical functioning, role limitation to physical problems, bodily pain, vitality, social functioning, role limitations to emotional problems, and mental health, as well as the impact of weight on the physical and psychosocial aspects of quality of life (IWQOL-Lite) and the burden of obesity-specific weight symptoms (WRSM) [21–25]. Participants completed all patient reported outcome measures (PROMs) before the first period of 8-week MICT, after the second period of 8-week HIIT or MICT, and after the third period of 8-week MICT. Finally, we assessed potential associations between maximal cardiorespiratory fitness and the different measures of HRQOL.

### The short form health survey (SF-36)

SF-36 (version 2.0) is a 36-item measure of generic HRQOL in which 35 questions are transformed into eight dimensions; physical functioning, role limitation to physical problems, bodily pain, general health, vitality, social functioning, role limitations to emotional problems, and mental health [21, 22]. Scores on all eight dimensions range from zero to 100, where higher scores represent better HRQOL. The SF-36 has been translated into Norwegian [26] and has demonstrated good psychometric properties in different medical condition [26–29], also in patients with severe obesity [30].

### The impact of weight on the physical and psychosocial aspects of quality of life (IWQOL-Lite)

IWQOL-Lite is a 31-item measure of weight-related quality of life [23]. Four domain scores were collected in this study (physical function, self-esteem, sexual life and public distress). Scores for all domains range from 0–100, with lower scores indicating greater impairment.

### The Weight-Related Symptom Measure (WRSM)

WRSM is a 20-item measure for the presence and distress of 20 weight-related symptoms [24, 25]. The symptoms are scored on a six-point Likert scale. The scale ranges from zero (does not bother at all) to six (bothers a very great deal) with higher scores indicating worse symptom distress.

### Maximal cardiorespiratory fitness (VO_2max_)

VO_2max_ [31] was expressed as liters per min (L*min^−1^) and was measured by use of Metalyser, Cortex 2 (Biophysik, Leipzig, Germany). The maximal cardiorespiratory fitness test was performed at baseline and weeks 8, 16, and 24; this as an individualized incremental treadmill test on a Woodway PPS 55 plus (Waukesha, Germany). Either the velocity was increased alternately by 0.5 km*h^−1^, or the inclination was increased by 1% every 30 s until voluntary exhaustion. The duration of the tests ranged from four to 10 min.

### Sample size and randomization.

The sample size was calculated based on the primary outcome (energy expenditure during exercise) in a recently published study [20]. Anticipating a relatively high initial drop-out rate, randomization was performed after the first exercise period, at the end of week 7 (Table 1). Patients completing the first 7-weeks were stratified into two groups, above or below baseline median of maximal cardiorespiratory fitness, and subsequently block-randomized into either the MICT or the HIIT/MICT-group, using block-sizes of four. The randomization was performed by the study biostatistician (MCS) using Stata (Version 14.2).

**Table 1:** Baseline characteristics

	MICT-group (n = 34)	HIIT/MICT-group (n = 37)
*Sex*		
Female	19 (56%)	20 (54%)
Male	15 (44%)	17 (46%)
*Age (years)*	44.2 (9.8)	43.3 (12.6)
*White ethnicity*	31 (91.2%)	37 (100%)
*Anthropometrics*		
Body weight (kg)	127 (23.9)	120 (20.2)
Body mass index (kg*m^−2^)	42.8 (5.3)	41.1 (5.1)
Waist circumference (cm)	123 (15.3)	119 (11.0)
Fat-free mass (kg)	70.1 (17.3)	68.0 (14.9)
Fat mass (kg)	56.8 (13.0)	51.7 (12.2)
*Education*^*#*^		
Above 13 years	19 (58%)	12 (34%)
Below 13 years	14 (42%)	23 (66%)
*Marital status*^¤^		
Married/living together	23 (68%)	23 (68%)
Single/divorced/widower	11 (32%)	11 (32%)
*Employment*^*#*^	28 (82%)	27 (77%)

Data presented as counts (%) or mean (SD). ^¤^; N = 34 MICT-group and 34 HIIT/MICT-group, ^#^; N = 33 MICT-group and 35 HIIT/MICT-group

### Statistical analyses

Descriptive statistics are presented as mean and standard deviation (SD) for continuous variables or counts and percentages for categorical variables. Independent sample t-test, or Fisher`s exact test as appropriate were used to analyzed differences between groups at baseline. Outcome measures collected over time were analyzed using linear mixed models with an unstructured correlation matrix to account for statistical dependencies as the same individuals were measured three times, at baseline, 16-week and 24-week. All randomly assigned patient data from baseline were analysed according to the intention-to-treat principle and to the per-protocol principle, with treatment, time and treatment-time interaction entered as the fixed effects. In the linear mixed models the fixed effects were taking any random baseline differences into account. The per-protocol analyses were used as a sensitivity analysis by excluding patients who withdrew, discontinued intervention, and/or attended fewer than 70% of prescribed exercise sessions. Confidence interval (CI) were used to quantify the uncertainty in the sample variables. Standardized effect sizes (ESs) were calculated in order to facilitate interpretation of within- and between-group differences [32]. ESs within groups were calculated as the mean changes in the various HRQOL dimension scores between week 24 and baseline divided by the SD of the respective baseline dimension of HRQOL. ESs between groups were calculated as the differences in mean changes in HRQOL scores between groups at 24-week divided by SDs from the baseline HRQOL dimension (Cohen`s d) [32, 33]. The ES was considered small (0.20- 0.49), moderate (0.50- 0.79) or large (0.80- ›) [32, 33]. The reliability of the questionnaires` dimensions was assessed with Cronbach`s alpha, and all measurements (SF-36, IWQOL-Lite and WRSM) had satisfactory reliability (Cronbach`s alpha values > 0.70). *P*-values < 0.05 were considered statistically significant. No correction for multiple testing was performed, as the results are exploratory. All statistical analyses were performed using the Statistical Package for Social Sciences (SPSS) version 23 (Chicago, IL).

## Results

**Fig. 2:**
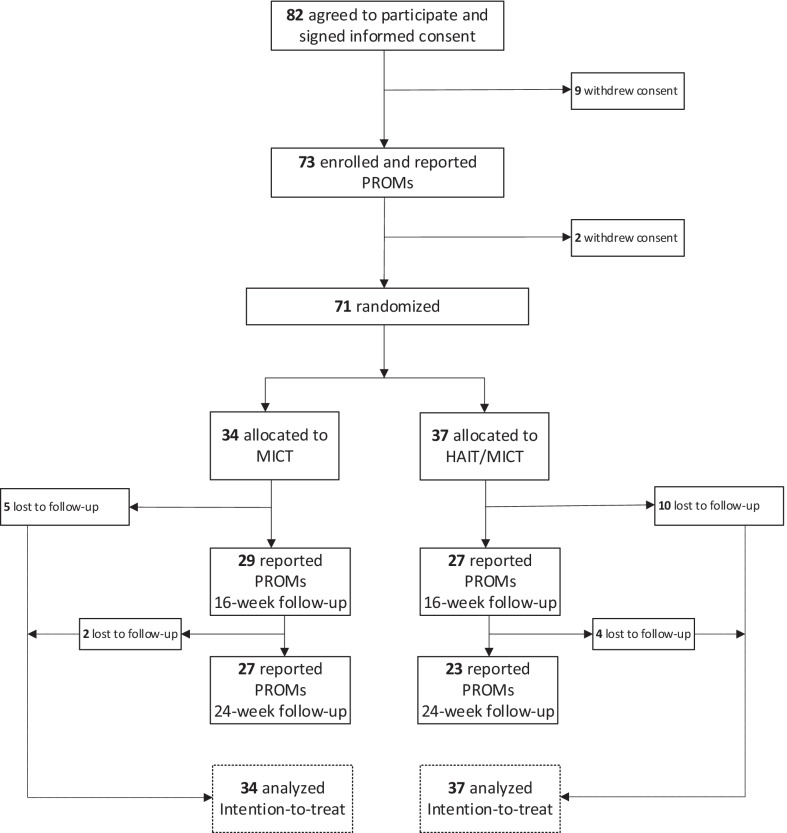
Flow chart

A total of 82 patients (56% females) underwent medical screening and agreed to participate, 11 withdrew consent, and 73 patients were initially enrolled in the study and reported PROMs. However, before randomization, 2 patients reporting PROMs withdrew their consent, leaving 71 patients (55% females) who had completed the first 8-week program to be allocated to either 16 weeks MICT (n = 34) or 8 weeks HIIT followed by 8 weeks MICT (n = 37) (Fig. 2). A total of 21 randomized patients (30%) were lost to follow-up at 24-week, leaving 50 patients who completed the 24-weeks intervention and reported PROMs (Fig. 2). The 71 randomized patients were included in the intention-to-treat analysis (MICT-group, n = 34; HIIT/MICT-group, n = 37).

The patients had a baseline mean (SD) age of 43.8 (11.3) years, BMI 41.9 (5.3) kg*m^−2^ and body weight of 123.3 (22.2) kg (Table 1). Further, 46% of the patients had higher education, 68% were married or living together, and 81% were at least part-time employed (Table 1). Baseline characteristics did not differ significantly between the groups.

Baseline characteristics between completers and non-completers were comparable (Additional file 3).

### Main outcome

**Fig. 3:**
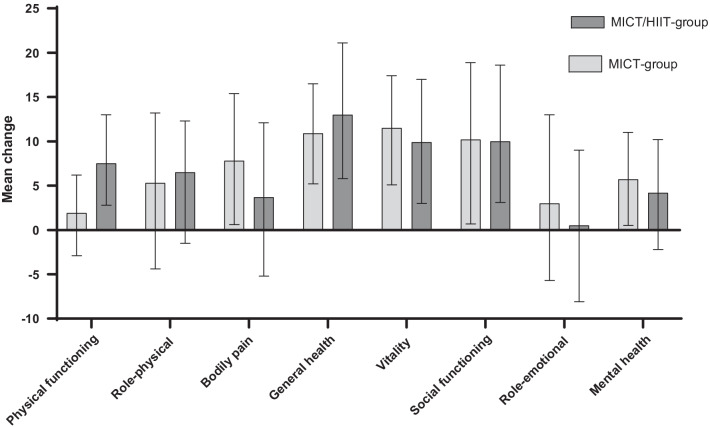
Mean change scores (% CI) on eight HRQL-dimensions from baseline to 24-week between groups

In the intention-to-treat analysis, general health scores increased moderately between baseline and 24-week follow-up in both the HIIT/MICT group and the MICT group, with a mean change of 13 (95% CI 6–21) points and 11 (95% CI 5–17) points, respectively (Table 2 and Fig. 3). Corresponding ESs were 0.7 and 0.6 (Table 2). However, there was no statistically significant difference between the groups (Table 2).

**Table 2:** Main and secondary outcomes (SF-36)

	MICT-group (n = 34)	*p*-value within group	Effect size	HIIT/MICT-group (n = 37)	*p*-value within group	Effect size	Between group differences (95% CI)	*p*-value between group	Effect size between group
*Main outcome*									
*General health*									
Baseline	52.6 (46.1, 59.1)	–		52.7 (46.4, 58.9)	–		–	–	
24-Week	63.5 (56.6, 70.5)	–		65.7 (58.5, 72.8)	–		− 2.1 (− 12.1, 7.8)	0.668	
Change from baseline to 24-week	10.9 (5.2, 16.5)	< 0.001	0.60	13.0 (5.8, 21.1)	0.001	0.67	− 2.1 (− 7.1, 11.3)	0.651	0.11
*Secondary outcomes*									
*Physical functioning*									
Baseline	75.3 (69.9, 80.7)	–		75.9 (70.8, 81.1)	–		–	–	
24-Week	77.2 (71.5, 82.8)	–		83.4 (77.7, 89.1)	–		− 6.2 (− 14.2, 1.8)	0.127	
Change from baseline to 24-week	1.9 (− 2.9, 6.2)	0.470	0.14	7.5 (2.8, 13.0)	0.004	0.43	− 5.6 (− 1.1, 12.2)	0.098	0.32
*Role-physical*									
Baseline	77.6 (69.6, 85.5)	–		79.4 (71.8, 87.0)	–		–	–	
24-Week	82.9 (75.4, 90.4)	–		85.9 (78.0, 93.8)	–		− 3.0 (− 13.9, 7.9)	0.585	
Change from baseline to 24-week	5.3 (− 4.4, 13.2)	0.317	0.23	6.5 (− 1.5, 12.3)	0.103	0.27	− 1.2 (− 11.2, 13.5)	0.852	0.05
*Bodily pain*									
Baseline	58.8 (51.4, 66.2)	–		64.0 (56.9, 71.1)	–		–	–	
24-Week	66.6 (58.5, 74.8)	–		67.7 (59.2, 76.1)	–		− 1.0 (− 12.8, 10.7)	0.864	
Change from baseline to 24-week	7.8 (0.6, 15.4)	0.036	0.38	3.7 (− 5.2, 12.1)	0.423	0.16	4.2 (− 15.3, 6.9)	0.451	0.18
*Vitality*									
Baseline	39.5 (33.7, 45.4)	–		42.9 (37.3, 48.5)	–		–	–	
24-Week	51.0 (44.1, 57.8)	–		52.8 (45.7, 59.9)	–		− 1.8 (− 11.7, 8.0)	0.711	
Change from baseline to 24-week	11.5 (5.1, 17.4)	0.001	0.68	9.9 (3.0, 17.0)	0.006	0.58	1.5 (− 10.7, 7.6)	0.736	0.09
*Social functioning*									
Baseline	74.3 (64.7, 83.9)	–		79.4 (70.2, 88.6)	–		–	–	
24-Week	84.5 (77.3, 91.7)	–		89.4 (82.0, 96.9)	–		− 4.9 (− 15.3, 5.4)	0.345	
Change from baseline to 24-week	10.2 (0.7, 18.9)	0.037	0.33	10.0 (3.1, 18.6)	0.007	0.40	0.2 (− 12.2, 11.8)	0.974	0.01
*Role-emotional*									
Baseline	81.1 (72.6, 89.6)	–		82.7 (74.5, 90.8)	–		–	–	
24-Week	84.1 (76.4, 91.9)	–		83.2 (75.1, 91.3)	–		1.0 (− 10.3, 12.2)	0.865	
Change from baseline to 24-week	3.0 (− 5.7, 13.0)	0.424	0.12	0.5 (− 8.1, 9.0)	0.915	0.02	2.5 (− 14.2, 9.2)	0.672	0.11
*Mental health*									
Baseline	68.7 (62.2, 75.1)	–		71.2 (65.1, 77.4)	–		–	–	
24-Week	74.4 (68.7, 80.0)	–		75.4 (69.5, 81.2)	–		− 1.0 (− 9.2, 7.1)	0.803	
Change from baseline to 24-week	5.7 (0.5, 11.0)	0.033	0.26	4.2 (− 2.2, 10.2)	0.197	0.29	1.5 (− 9.2, 6.2)	0.693	0.10

Estimated mean (95% CI). Effect size, Cohen’s d

### Secondary outcomes

The vitality and social functioning scores increased in both the HIIT/MICT group and the MICT group, with no statistically significant between-group difference (Table 2 and Fig. 3). The remaining dimensions (physical functioning, role limitation to physical problems, bodily pain, role limitations to emotional problems, and mental health) did not change significantly between groups (Table 2 and Fig. 3).

The physical function and self-esteem scores based on IWQOL-Lite increased in both exercise groups, with no differences between the groups (Additional file 1). The tiredness, back pain and physical stamina scores based on WRSM decreased similarly in both groups (Additional file 1).

The per-protocol results (sensitivity analyses) mirrored the ITT results (Additional file 4).

### Associations between maximal cardiorespiratory fitness and HRQOL (SF-36)

The changes in maximal cardiorespiratory fitness were significant and negatively associated with changes in general health (Pearson`s r = -0.30) (n = 71). Both groups increased the maximal cardiorespiratory fitness from baseline to 24 week with no statistically significant different between the groups, as recently published [20] (Additional file 2). No significant associated between changes in body weight and changes in general health were found (Pearson`s r = -0.19).

## Discussion

### Brief synopsis of key findings

In contrast to our hypothesis, treatment-seeking patients with severe obesity who were randomized to a 24-week combined MICT and HIIT program did not show greater improvement in the general health dimension of SF-36 compared with those who completed a 24-week MICT program. However, the general health scores increased significantly in both groups, with a moderate effect size. The vitality and social functioning scores of SF-36, and the physical function and self-esteem scores of IWQOL-Lite increased similarly in both groups, while tiredness, back pain and physical stamina scores of WRSM decreased similarly in both groups.

### Possible mechanisms and explanations

As previously reported [20], maximal cardiorespiratory fitness improved similarly in both groups. A previous cross-sectional study in patients with severe obesity preparing for bariatric surgery [7] found a correlation between submaximal cardiorespiratory fitness (treadmill time) and physical function, role physical, bodily pain, and mental health, with no observed correlation in general health. However, this study [7] did not use maximal testing with direct assessment of maximal cardiorespiratory fitness. In a cross-sectional study [8] of normal- to overweight patients with schizophrenia (n = 33) that used an indirect assessment of maximal cardiorespiratory fitness (VO_2max_, L*min^−1^), this was found to be significantly correlated with general health (Pearson`s r = 0.53), physical function, and social function [8]. One could speculate that increased maximal cardiorespiratory fitness may cause increased general health.

In a previous randomized study intervention with inactive individuals (BMI 33.3–64.8 kg*m^−2^) performing 16 weeks of HIIT (85–95% of HR_max_) or MICT (40–55% of HR_max_) no correlation between changes in HRQOL and changes in maximal cardiorespiratory fitness were observed [19]. However, these negative findings were based on changes in the mental and physical summary scores of SF-36. Importantly, these scores may not capture essential changes in the single dimensions. Accordingly, the interpretation of these summary scores has been debated [34, 35]. The changes in maximal cardiorespiratory fitness in our present study was negatively associated with changes in general health. Perhaps a change in maximal cardiorespiratory fitness of greater than the 10% that is found in our study is needed to affect general health. Importantly, exercise, independent of intensity, increased maximal cardiorespiratory fitness and seemed to impact general health in both the present and a previous randomized study [19].

In general, weight loss improves HRQOL [1]. However, the weight loss in the present study did not correlate significantly with changes in the general health dimension. This lack of coherence between changes in weight loss and general health may be caused by the relatively small weight loss. Our findings support those from a recent study showing that 16 weeks of HIIT or MICT and a weight loss of 2–3 kg did not correlate with changes in summary scores of HRQOL, respectively [19].

Therefore, based on our results and the previous intervention study [19], it seems that both moderate and high intensity exercise confer some HRQOL benefits in patients with severe obesity, together with increased maximal cardiorespiratory fitness and weight loss.

Our study used a generic measure of HRQoL (SF-36) and was complementary with an obesity-specific measure of HRQOL (IWQOL-Lite) and a symptom measure of obesity (WRSM). The within group changes provided the most useful information about the patients’ experience of weight-related quality of life and symptoms. Exercise was, overall, associated with improved IWQOL-Lite scores independent of exercise intensity (Additional file 1). Despite no statistically significant differences between groups, a numerical greater within group changes in WRSM scores was found in the HIIT/MICT group, compared with the MICT group (Additional file 1). Despite that, high intensity is regarded as a negative exercise method for a sedentary and overweight population, because of negative changes in the affective responses from exercise with high intensity [36–38]. The somewhat surprising findings may potentially be explained by adaptation to higher intensities and thus greater self-efficacy [39, 40].

### Strengths and limitations

This study has a number of limitations. First, the selection of participants who were particularly motivated to increase physical activity before undergoing a conventional weight loss may reduce the generalizability of our results. Second, only treatment-seeking, predominantly white patients with severe obesity participated in the present study, thus limiting the generalizability to other populations. Third, as is usual for this kind of exercise study, the dropout rate was relatively high. [13, 18, 40–42], which might have biased the results. Fourth, the proportion of patients who completed the exercise program was considerably lower in the HIIT/MICT group than MICT, which might have biased the results. Fifth, the sample size was based on the primary outcome measured (energy expenditure during exercise) in the recent published article [20], and not the HRQOL measures.

This study is strengthened by its randomized controlled design, and the generally accepted and validated methodology measuring generic- and obesity specific health related quality of life, weight-related symptoms, and maximal cardiorespiratory fitness. The relatively long exercise intervention (6 months) for patients with obesity is also a strength. However, the study may only be generalizable to patients with severe obesity who are motivated and able to implement exercise over time. Future studies should seek to include a more diverse sample population.

## Conclusions

Patients with severe obesity who completed a 24-week HIIT/MICT program did not improve general health to a greater extent than those completing a 24-week MICT program. No differences were found in the other seven dimensions of SF-36 scores, in obesity-specific HRQOL scores, or in weight-related symptoms. However, independent of exercise intensity, general health improved in both groups after 24-week exercise.

## Implications

From a practical point of view, it might be appropriate to inform patients with severe obesity that aerobic exercise may improve general health independently of exercise intensity.

## Supplementary Information

**Additional file 1.** IWOOL-Lite and WRSM.

**Additional file 2.** Maximal cardiorespiratory fitness and body composition.

**Additional file 3.** Drop-outs.

**Additional file 4.** Per-protocol analysis (sensitivity analysis).

## Data Availability

The protocol and datasets used and/or analyzed during the current study are available from the corresponding author (Jarle.berge@siv.no) on reasonable request. The datasets supporting the conclusions of this article are included within the article and its additional files. The primary article for this randomized controlled trial: https://doi.org/10.1002/oby.23078

## Abbreviations

MICT: Moderate-intensity continuous training program.
HIIT: High-intensity interval training program.
HIIT/MICT: High-intensity interval training program combined with moderate-intensity continuous training program.
SF-36: The Short form health survey.
IWQOL-Lite: The impact of weight on the physical and psychosocial aspects of quality of life.
WRSM: The burden of obesity-specific weight symptoms.
HRQOL: Patients health related quality of life.
BMI: Body mass index.
BPM: Beats per minutes.
HR_max_: Heart rate maximum.
PROMs: Patient reported outcome measures.
VO_2max_: Maximal cardiorespiratory fitness.
SD: Standard deviation.
CI: Confidence interval.
ESs: Effect sizes
