# Supplementary material for: Effect of aerobic exercise intensity on health-related quality of life in severe obesity: a randomized controlled trial
Source: Health Qual Life Outcomes. 2022 Feb 24;20:34. doi: 10.1186/s12955-022-01940-y (PMC8876087; doi:10.1186/s12955-022-01940-y)
Supplement: Supplementary file 1 — Additional file 1. IWOOL-Lite and WRSM. [file 12955_2022_1940_MOESM1_ESM.pdf]

Additional file 1

	MICT-group (n=34)	p-value within group	Effect size	HIIT/MICT-group (n=37)	p-value within group	Effect size	Between group differences (95%CI)	p-value between group	Effect size between group
Impact of Weight on Quality of Life (IWQOL-Lite)									
**Physical function**									
Baseline	56.9 (49.6, 64.2)	-		56.0 (48.9, 63.0)	-		-	-	
24-week	66.5 (59.1, 74.0)	-		68.6 (61.2, 76.1)	-		-2.1 (-12.6, 8.4)	0.690	
Change from baseline to 24-week	9.6 (4.8, 14.2)	<0.001	0.41	12.6 (7.1, 18.8)	<0.001	0.65	-3.0 (-4.1, 10.2)	0.401	0.15
**Self-Esteem**									
Baseline	49.8 (39.7, 59.9)	-		48.1 (38.4, 57.7)	-		-	-	
24-week	57.2 (46.6, 67.8)	-		59.2 (48.9, 69.6)	-		-2.0 (-16.8, 12.7)	0.783	
Change from baseline to 24-week	7.4 (3.0, 11.4)	0.002	0.24	11.1 (5.8, 16.5)	<0.001	0.39	-3.7 (-2.8, 10.4)	0.258	0.13
**Sexual life**									
Baseline	65.4 (55.5, 75.3)	-		70.9 (61.5, 80.4)	-		-	-	
24-week	69.4 (61.1, 77.8)	-		83.1 (74.7, 91.4)	-		-13.7 (-25.4, -1.8)	0.024	
Change from baseline to 24-week	4.0 (-2.5, 9.1)	0.256	0.13	12.2 (4.6, 19.9)	0.003	0.46	-8.1 (-1.2, 17.4)	0.086	0.30
**Public distress**									
Baseline	71.3 (63.5, 79.1)	-		77.0 (69.5, 84.5)	-		-	-	
24-week	69.7 (61.2, 78.2)	-		81.5 (73.0, 90.0)	-		-11.8 (-23.8, 0.2)	0.054	
Change from baseline to 24-week	-1.6 (-7.8, 2.2)	0.267	0.08	4.5 (-0.6, 10.7)	0.076	0.03	-6.1 (-1.6, 13.8)	0.116	0.25
Weight related symptom measure (WRSM)									
**Shortness of breath**									
Baseline	2.4 (1.9, 3.0)	-		2.2 (1.7, 2.7)	-		-	-	
24-week	1.8 (1.2, 2.4)	-		1.6 (1.0, 2.2)	-		0.2 (-0.7, 1.0)	0.718	
Change from baseline to 24-week	-0.6 (-1.2, -0.1)	0.017	0.40	-0.6 (-1.0, 0.0)	0.052	0.40	0.1 (-0.6, 0.9)	0.733	0.07
**Tiredness**									
Baseline	3.2 (2.7, 3.8)	-		3.2 (2.6, 3.7)	-		-	-	
24-week	2.6 (2.0, 3.2)	-		2.2 (1.6, 2.8)	-		0.3 (-1.1, 0.6)	0.499	
Change from baseline to 24-week	-0.6 (-1.2, -0.1)	0.032	0.40	-1.0 (-1.6, -0.3)	0.005	0.56	0.4 (-0.5, 1.2)	0.389	0.22
**Sleep problems**									
Baseline	2.0 (1.4, 2.7)	-		2.2 (1.6, 2.9)	-			-	
24-week	2.0 (1.4, 2.5)	-		1.6 (1.1, 2.2)	-		0.3 (-0.5, 1.1)	0.424	

Change from baseline to 24-week	0.0 (-0.2, 0.4)	0.571	0.00	-0.6 (-1.1, -0.1)	0.029	0.29	0.6 (-1.1, 0.1)	0.074	0.29
**Sensitivity to cold**									
Baseline	0.9 (0.5, 1.4)	-		0.7 (0.2, 1.2)	-		-	-	
24-week	0.8 (0.3, 1.2)	-		0.6 (0.1, 1.1)	-		0.2 (-0.4, 0.9)	0.469	
Change from baseline to 24-week	-0.1 (-0.4, 0.2)	0.406	0.06	-0.1 (-0.6, 0.3)	0.556	0.09	0.0 (-0.6, 0.6)	0.990	0.00
**Increased thirst**									
Baseline	1.6 (1.0, 2.1)	-		1.4 (0.9, 1.9)	-		-	-	
24-week	1.3 (0.8, 1.8)	-		0.9 (0.4, 1.4)	-		0.4 (-0.3, 1.1)	0.269	
Change from baseline to 24-week	-0.3 (-0.8, 0.2)	0.246	0.19	-0.5 (-1.1, 0.1)	0.014	0.33	0.2 (-0.9, 0.5)	0.533	0.13
**Increased irritability**									
Baseline	1.8 (1.2, 2.4)	-		1.8 (1.3, 2.4)	-		-	-	
24-week	1.5 (0.9, 2.0)	-		0.9 (0.3, 1.5)	-		0.5 (-0.3, 1.3)	0.195	
Change from baseline to 24-week	-0.3 (-0.8, 0.2)	0.244	0.20	-0.9 (-1.5, -0.3)	0.005	0.50	0.6 (-1.3, 0.2)	0.169	0.33
**Back pain**									
Baseline	2.1 (1.4, 2.7)	-		2.4 (1.7, 3.0)	-		-	-	
24-week	1.3 (0.8, 1.9)	-		1.6 (1.0, 2.1)	-		-0.2 (-1.0, 0.6)	-	
Change from baseline to 24-week	-0.8 (-1.1, -0.3)	0.002	0.50	-0.8 (-1.5, -0.2)	0.018	0.38	0.1 (-0.8, 0.7)	-	0.05
**Frequent urination**									
Baseline	1.8 (1.2, 2.4)	-		1.2 (0.7, 1.8)	-		-	-	
24-week	1.5 (1.0, 2.1)	-		0.9 (0.4, 1.5)	-		0.6 (-0.2, 1.4)	0.128	
Change from baseline to 24-week	-0.3 (-0.7, 0.2)	0.227	0.18	-0.3 (-0.9, 0.3)	0.268	0.19	0.0 (-0.7, 0.7)	0.974	0.00
**Pain in the joints**							-	-	
Baseline	2.8 (2.2, 3.4)	-		2.4 (1.8, 3.0)	-		-	-	
24-week	2.6 (1.9, 3.4)	-		1.9 (1.1, 2.7)	-		0.7 (-0.4, 1.8)	0.195	
Change from baseline to 24-week	-0.2 (-0.7, 0.4)	0.585	0.10	-0.5 (-1.4, 0.3)	0.188	0.29	0.3 (-1.3, 0.7)	0.564	0.18
**Water retention**									
Baseline	2.2 (1.7, 2.8)	-		1.3 (0.5, 1.7)	-		-	-	
24-week	1.9 (1.3, 2.5)	-		1.0 (0.4, 1.6)	-		0.9 (0.0, 1.7)	0.040	
Change from baseline to 24-week	-0.3 (-0.8, 0.1)	0.153	0.17	-0.3 (-0.7, 0.2)	0.248	0.21	0.0 (-0.6, 0.7)	0.764	0.00
**Foot problems**									
Baseline	1.8 (1.3, 2.3)	-		1.2 (0.6, 1.7)	-		-	-	
24-week	1.8 (1.2, 2.3)	-		1.1 (0.5, 1.7)	-		0.7 (-0.1, 1.5)	0.090	
Change from baseline to 24-week	0.0 (-0.4, 0.4)	0.982	0.00	-0.1 (-0.8, 0.5)	0.700	0.07	0.1 (-0.8, 0.7)	0.848	0.07
**Sensitivity to heat**									
Baseline	1.6 (1.0, 2.3)	-		1.4 (0.8, 2.0)	-		-	-	
24-week	1.3 (0.7, 1.9)	-		1.1 (0.4, 1.7)	-		0.2 (-0.6, 1.1)	0.600	

Change from baseline to 24-week	-0.3 (-0.9, 0.2)	0.211	0.16	-0.3 (-1.0, 0.3)	0.275	0.17	0.0 (-0.8, 0.9)	0.923	0.00
**Snoring**									
Baseline	2.6 (1.9, 3.4)	-		2.4 (1.7, 3.1)	-		-	-	
24-week	2.0 (1.3, 2.7)	-		1.6 (0.9, 2.3)	-		0.4 (-0.6, 1.4)	0.397	
Change from baseline to 24-week	-0.6 (-1.4, 0.1)	0.076	0.27	-0.8 (-1.4, -0.2)	0.015	0.38	0.2 (-1.1, 0.8)	0.716	0.10
**Increased appetite**									
Baseline	2.0 (1.4, 2.5)	-		1.7 (1.2, 2.2)	-		-	-	
24-week	1.9 (1.3, 2.5)	-		1.1 (0.5, 1.7)	-		0.8 (-0.0, 1.6)	0.054	
Change from baseline to 24-week	-0.1 (-0.7, 0.6)	0.836	0.06	-0.6 (-1.3, 0.0)	0.065	0.38	0.5 (-1.4, 0.6)	0.227	0.31
**Leakage of urine**									
Baseline	0.7 (0.3, 1.2)	-		0.4 (0.0, 0.9)	-		-	-	
24-week	0.6 (0.2, 1.1)	-		0.6 (0.1, 1,1)	-		0.0 (-0.6, 0.7)	0.829	
Change from baseline to 24-week	-0.1 (-0.3, 0.1)	0.330	0.07	0.2 (-0.3, 0.5)	0.610	0.20	-0.2 (-0.2, 0.6)	0.247	0.20
**Lightheadeness**									
Baseline	0.7 (0.3, 1.2)	-		0.9 (0.5, 1.3)	-		-	-	
24-week	0.8 (0.4, 1.3)	-		0.6 (0.1, 1.0)	-		0.2 (-0.4, 0.9)	0.413	
Change from baseline to 24-week	0.1 (-0.2, 0.5)	0.416	0.083	-0.3 (-0.7, -0.0)	0.038	0.200	0.4 (-0.9, 0.0)	0.074	0.27
**Increased sweating**									
Baseline	1.5 (0.9, 2.2)	-		1.7 (1.1, 2.3)	-		-	-	
24-week	1.2 (0.7, 1.7)	-		1.1 (0.5, 1.6)	-		0.1 (-0.6, 0.9)	0.636	
Change from baseline to 24-week	-0.3 (-0.8, 0.2)	0.250	0.16	-0.6 (-1.1, -0.2)	0.007	0.33	0.3 (-1.0, 0.4)	0.360	0.17
**Loss of sexual desire**									
Baseline	1.7 (1.1, 2.3)	-		1.6 (1.0, 2.2)	-		-	-	
24-week	1.6 (1.1, 2.1)	-		0.9 (0.4, 1.4)	-		0.7 (-0.0, 1.5)	0.059	
Change from baseline to 24-week	-0.1 (-0.7, 0.5)	0.848	0.06	-0.7 (-1.3, -0.1)	0.021	0.39	0.6 (-1.4, 0.2)	0.148	0.33
**Physical stamina**									
Baseline	3.1 (2.5, 3.7)	-		2.9 (2.3, 3.5)	-		-	-	
24-week	1.7 (1.1, 2.3)	-		1.2 (0.6, 1.9)	-		0.5 (-0.4, 0.7)	0.236	
Change from baseline to 24-week	-1.4 (-2.1, -0.6)	0.001	0.82	-1.7 (-2.4, -1.0)	<0.001	0.90	0.3 (-1.4, 0.7)	0.522	0.16
**Skin irritation**									
Baseline	1.2 (0.7, 1.7)	-		0.8 (0.3, 1.2)	-		-	-	
24-week	1.2 (0.7, 1.7)	-		0.3 (-0.3, 0.8)	-		0.9 (0.2, 1.7)	0.017	
Change from baseline to 24-week	0.0 (-0.7, 0.7)	0.989	0.00	0.5 (-0.9, -0.1)	0.018	0.42	-0.5 (-1.3, 0.3)	0.243	0.42
