# Supplementary material for: Effect of aerobic exercise intensity on health-related quality of life in severe obesity: a randomized controlled trial
Source: Health Qual Life Outcomes. 2022 Feb 24;20:34. doi: 10.1186/s12955-022-01940-y (PMC8876087; doi:10.1186/s12955-022-01940-y)
Supplement: Supplementary file 2 — Additional file 2. Maximal cardiorespiratory fitness and body composition. [file 12955_2022_1940_MOESM2_ESM.pdf]

Additional file 2

	MICT-group	p-value within group	HIIT/MICT-group	p-value within group	Between group differences (95%CI)	p-value between group
**Maximal cardiorespiratory fitness (VO_{2max}, $L*min^{-1}$)**						
Intention-to-treat analysis	(n=34)		(n=37)			
Baseline	3.18 (2.92, 3.44)	-	3.07 (2.85, 3.29)	-	-	-
24-week	3.45 (3.20, 3.71)	-	3.40 (3.13, 3.67)	-	0.07 (-0.28, 0.44)	0.681
Change from baseline to 24-week	0.27 (0.20, 0.34)	<0.001	0.32 (0.18, 0.47)	<0.001	-0.03 (-0.11, 0.18)	0.644
Percent change	9.4	-	9.7	-	-	-
**Body mass index ($kg*m^{-2}$)**						
Intention-to-treat analysis	(n=34)		(n=37)			
Baseline	42.8 (40.9, 44.6)	-	41.1 (39.4, 42.9)	-	-	-
24-week	42.4 (40.4, 44.4)	-	39.8 (37.9, 41.7)	-	2.6 (-0.2, 5.4)	0.063
Change from baseline to 24-week	-0.4 (-0.9, 0.2)	0.181	-1.4 (-2.0, -0.7)	<0.001	0.9 (-1.8, -0.1)	0.025
Percent change	0.9	-	3.2	-	-	-
**Body weight (kg)**						
Intention-to-treat analysis	(n=34)		(n=37)			
Baseline	127 (119.7, 135)	-	120 (113, 127)	-	-	-
24-week	126 (118, 134)	-	116 (108, 124)	-	10.0 (-1.0, 21.0)	0.074
Change from baseline to 24-week	-1.3 (-2.9, 0.5)	0.169	-3.8 (-5.5, -1.8)	<0.001	2.5 (-4.9, -0.1)	0.046
Percent change	1.0	-	3.2	-	-	-
**Waist circumference (cm)**						
Intention-to-treat analysis	(n=34)		(n=37)			
Baseline	123 (118, 127)	-	119 (115, 123)	-	-	-
24-week	118 (113, 123)	-	113 (108, 118)	-	4.6 (-2.1, 11.4)	0.177
Change from baseline to 24-week	-4.9 (-6.4, -3.3)	<0.001	-6.0 (-7.7, -4.2)	<0.001	1.1 (-3.2, 1.0)	0.299
Percent change	4.0	-	5.0	-	-	-
**Fat-free Mass (kg)**						
Intention-to-treat analysis	(n=34)		(n=37)			
Baseline	70.1 (64.6, 75.6)	-	68.0 (62.7, 73.3)	-	-	-
24-week	70.2 (64.8, 75.7)	-	67.0 (61.8, 72.2)	-	3.2 (-4.3, 10.8)	0.397
Change from baseline to 24-week	0.1 (-1.0, 1.3)	0.387	-1.0 (-1.6, -0.2)	0.019	0.9 (-2.5, 0.2)	0.095
Percent change	0.1	-	1.5	-	-	-
