# Supplementary material for: Effect of aerobic exercise intensity on health-related quality of life in severe obesity: a randomized controlled trial
Source: Health Qual Life Outcomes. 2022 Feb 24;20:34. doi: 10.1186/s12955-022-01940-y (PMC8876087; doi:10.1186/s12955-022-01940-y)
Supplement: Supplementary file 3 — Additional file 3. Drop-outs. [file 12955_2022_1940_MOESM3_ESM.pdf]

Additional file 3. Drop-outs

	**MICT-group (n=27)**	**MICT-group, drop-outs (n=7)**	**p-value**	**HIIT/MICT-group (n=23)**	**HIIT/MICT-group, drop-outs (n=14)**	**p-value**
**Sex**						
Female	11 (46 %)	8 (80 %)	0.128	14 (61 %)	6 (43 %)	0.328
Male	13 (54 %)	2 (20 %)		9 (40 %)	8 (57 %)	
**Age (years)**	45.6 (10.1)	38.1 (8.6)	0.059	45.5 (12.4)	39.7 (12.8)	0.184
**Anthropometrics**						
Body weight (kg)	127 (18.3)	121 (37.1)	0.501	115 (20.6)	126 (18.3)	0.116
Body mass index (kg/m^2)	42.7 (4.6)	41.5 (7.8)	0.551	40.7 (5.8)	41.9 (4.0)	0.511
Waist circumference (cm)	123 (12.6)	117 (22.0)	0.263	118 (11.7)	121 (9.8)	0.355
Fat-free mass (kg)	71.8 (16.8)	61.3 (17.1)	0.112	64.8 (13.8)	73.3 (15.7)	0.094
Fat mass (kg)	55.1 (9.6)	59.6 (20.0)	0.368	50.8 (11.7)	53.2 (13.3)	0.580
**Cardiorespiratory fitness**						
VO_{2max} (L*min^{-1})	3.2 (0.7)	2.9 (0.8)	0.202	3.1 (0.6)	3.1 (0.7)	0.964
**Education**[#]						
Above 13 years	15 (58 %)	4 (57 %)	1.000	10 (44 %)	2 (17 %)	0.149
Below 13 years	11 (42 %)	3 (43 %)		13 (57 %)	10 (83 %)	
**Marital status**[¤]						
Married/living together	19 (70 %)	4 (57 %)	0.656	16 (70 %)	7 (64 %)	1.000
Single/divorced/widower	8 (30 %)	3 (43 %)		7 (30 %)	4 (36 %)	
**Employment**[#]	24 (89 %)	4 (57 %)	0.086	10 (44 %)	2 (17 %)	0.149

Data presented as numbers (%) or mean (SD), independent sample t-test, or Fisher`s exact test as appropriate. [#]; N= 33 MICT-group and 35 HIIT/MICT-group. [¤]; N= 34 MICT-group and 34 HIIT/MICT-group
