# Supplementary material for: Effect of aerobic exercise intensity on health-related quality of life in severe obesity: a randomized controlled trial
Source: Health Qual Life Outcomes. 2022 Feb 24;20:34. doi: 10.1186/s12955-022-01940-y (PMC8876087; doi:10.1186/s12955-022-01940-y)
Supplement: Supplementary file 4 — Additional file 4. Per-protocol analysis (sensitivity analysis). [file 12955_2022_1940_MOESM4_ESM.pdf]

Additional file 4. Per-protocol analysis (sensitivity analysis)

	MICT-group	p-value within group	HIIT/MICT-group	p-value within group	Between group differences (95%CI)	p-value between group
**SF-36**						
**General health score**						
Per-protocol analysis	(n=24)		(n=16)			
Baseline	55.8 (48.3, 63.3)	-	58.6 (49.5, 67.8)	-	-2.8 (-14.6, 9.0)	0.636
16 weeks	62.8 (55.2, 70.5)	-	74.7 (65.3, 84.1)	-	-11.9 (-24.0, 0.3)	0.055
24-week	67.4 (60.2, 74.6)	-	72.5 (63.6, 81.5)	-	-5.1 (-16.6, 6.4)	0.371
Change from baseline to 24-week	11.6 (5.0, 18.1)	0.001	13.9 (3.5, 23.8)	0.012	2.3 (-8.7, 13.4)	0.670
**Physical functioning**						
Per-protocol analysis	(n=24)		(n=16)			
Baseline	78.5 (72.5, 84.6)	-	80.0 (72.6, 87.4)	-	-1.5 (-11.0, 8.1)	0.759
16 weeks	83.3 (78.5, 88.2)	-	86.9 (80.9, 92.8)	-	-3.5 (-11.2, 4.1)	0.355
24-week	81.7 (76.7, 86.6)	-	89.1 (83.0, 95.3)	-	-7.5 (-15.4, 0.4)	0.063
Change from baseline to 24-week	3.2 (-1.2, 7.4)	0.147	9.1 (2.3, 15.7)	0.012	-6.0 (-1.3, 13.3)	0.104
**Role-physical**						
Per-protocol analysis	(n=24)		(n=16)			
Baseline	85.9 (77.0, 94.8)	-	82.4 (71.5, 93.3)	-	3.5 (-10.6, 17.6)	0.616
16 weeks	85.9 (78.8, 93.1)	-	92.6 (83.9, 101.3)	-	-6.6 (-17.9, 4.6)	0.240
24-week	89.6 (83.1, 96.1)	-	91.6 (83.5, 99.6)	-	-2.0 (-12.3, 8.3)	0.698
Change from baseline to 24-week	3.7 (-3.8, 11.1)	0.320	9.2 (-4.3, 22.3)	0.169	-5.5 (-8.0, 19.1)	0.416
**Bodily pain**						
Per-protocol analysis	(n=24)		(n=16)			
Baseline	62.8 (53.4, 72.2)	-	67.2 (55.7, 78.7)	-	-4.4 (-19.2, 10.5)	0.553
16 weeks	64.1 (55.1, 73.0)	-	65.3 (54.4, 76.3)	-	-1.2 (-15.4, 12.9)	0.861
24-week	71.8 (63.4, 80.2)	-	71.7 (61.3, 82.1)	-	0.1 (-13.2, 13.5)	0.986
Change from baseline to 24-week	9.0 (1.0, 17.0)	0.030	4.5 (-7.8, 17.0)	0.441	4.5 (-18.2, 9.2)	0.509
**Vitality score**						
Per-protocol analysis	(n=24)		(n=16)			
Baseline	42.7 (36.1, 49.4)	-	44.9 (36.8, 53.1)	-	-2.2 (-12.7, 8.3)	0.672
16 weeks	54.4 (48.1, 60.7)	-	56.1 (48.6, 63.9)	-	-1.8 (-11.8, 8.1)	0.712
24-week	55.5 (48.4, 62.6)	-	59.0 (50.2, 67.9)	-	-3.6 (-14.9, 7.8)	0.529
Change from baseline to 24-week	12.8 (6.0, 19.5)	0.001	14.1 (5.0, 23.3)	0.005	-1.4 (-9.5, 12.2)	0.802
**Social functioning score**						
Per-protocol analysis	(n=24)		(n=16)			
Baseline	80.7 (70.5, 91.0)	-	80.5 (67.9, 93.0)	-	0.3 (-16.0, 16.5)	0.974
16 weeks	93.2 (87.3, 99.1)	-	92.2 (85.0, 99.4)	-	1.0 (-8.3, 10.4)	0.823
24-week	91.1 (84.1, 98.2)	-	89.8 (81.1, 98.5)	-	1.3 (-9.8, 12.5)	0.812
Change from baseline to 24-week	10.4 (2.7, 18.1)	0.010	9.3 (0.5, 18.2)	0.039	1.1 (-12.6, 10.5)	0.854
**Role-emotional score**						
Per-protocol analysis	(n=24)		(n=16)			
Baseline	89.9 (81.3, 98.5)	-	84.9 (74.4, 95.4)	-	5.0 (-8.5, 18.6)	0.458
16 weeks	92.7 (85.6, 99.8)	-	93.2 (84.5, 101.9)	-	-0.5 (-11.7, 10.7)	0.926
24-week	90.6 (82.9, 98.3)	-	89.2 (76.7, 98.8)	-	1.4 (-10.9, 13.7)	0.821
Change from baseline to 24-week	0.7 (-7.5, 8.9)	0.862	4.3 (-3.5, 12.1)	0.261	-3.7 (-7.9, 15.2)	0.527
**Mental health score**						
Per-protocol analysis	(n=24)		(n=16)			
Baseline	75.0 (67.8, 82.2)	-	67.8 (59.0, 76.7)	-	7.2 (-4.2, 18.6)	0.210
16 weeks	78.3 (72.6, 84.1)	-	77.8 (70.8, 84.8)	-	0.5 (-8.6, 9.6)	0.908
24-week	78.3 (71.8, 84.9)	-	74.7 (66.5, 82.8)	-	3.7 (-6.8, 14.2)	0.484
Change from baseline to 24-week	3.3 (-2.2, 8.9)	0.227	6.9 (-1.3, 15.3)	0.090	-3.5 (-5.7, 12.8)	0.445

**IWQOL-Lite**						
**Physical function**						
Per-protocol analysis	(n=24)		(n=16)			
Baseline	60.1 (51.8, 68.5)	-	60.8 (50.6, 71.0)	-	-0.7 (-13.9, 12.6)	0.920
16 weeks	69.3 (61.8, 76.8)	-	75.7 (66.5, 84.9)	-	-6.4 (-18.3, 5.5)	0.284
24-week	70.5 (62.3, 78.7)	-	75.6 (65.6, 85.7)	-	-5.2 (-18.2, 7.8)	0.424
Change from baseline to 24-week	10.4 (5.1, 15.6)	<0.001	14.8 (7.1, 22.6)	0.001	-4.5 (-4.2, 13.2)	0.298
**Self-Esteem**						
Per-protocol analysis	(n=24)		(n=16)			
Baseline	58.2 (46.3, 70.1)	-	51.1 (36.6, 65.7)	-	7.1 (-11.7, 25.9)	0.451
16 weeks	63.1 (51.6, 74.6)	-	57.1 (43.1, 71.4)	-	5.7 (-12.4, 23.9)	0.527
24-week	65.0 (52.6, 77.5)	-	59.9 (44.6, 75.2)	-	5.1 (-14.6, 24.8)	0.602
Change from baseline to 24-week	6.8 (2.1, 11.6)	0.006	8.8 (3.3, 14.5)	0.004	-1.9 (-5.3, 9.2)	0.587
**Sexual life**						
Per-protocol analysis	(n=24)		(n=16)			
Baseline	72.1 (60.8, 83.4)	-	69.9 (56.1, 83.8)	-	2.2 (-15.6, 20.1)	0.803
16 weeks	77.3 (67.8, 86.9)	-	78.9 (67.2, 90.6)	-	-1.6 (-16.7, 13.6)	0.835
24-week	76.3 (67.1, 85.5)	-	83.0 (71.6, 94.4)	-	-6.7 (-21.4, 8.0)	0.360
Change from baseline to 24-week	4.2 (-1.5, 9.8)	0.141	13.1 (2.9, 23.0)	0.015	-8.9 (-1.4, 19.3)	0.088
**Public distress**						
Per-protocol analysis	(n=24)		(n=16)			
Baseline	78.3 (70.4, 86.3)	-	78.4 (68.7, 88.2)	-	-0.1 (-12.7, 12.5)	0.987
16 weeks	79.6 (72.0, 87.1)	-	84.1 (74.8, 93.3)	-	-4.5 (-16.4, 7.4)	0.452
24-week	76.9 (68.3, 85.5)	-	81.8 (71.2, 92.4)	-	-4.9 (-18.6, 8.8)	0.472
Change from baseline to 24-week	1.4 (-7.2, 4.3)	0.605	3.4 (-4.1, 10.4)	0.372	-4.8 (-4.1, 13.7)	0.282
Percent change	1.8	-	4.3	-	-	-
**WRSM**						
**Shortness of breath**						
Per-protocol analysis	(n=24)		(n=16)			
Baseline	2.1 (1.6, 2.7)	-	1.8 (1.1, 2.5)	-	0.3 (-0.6, 1.2)	0.483
16 weeks	1.6 (1.0, 2.2)	-	1.0 (0.3, 1.7)	-	0.6 (-0.4, 1.5)	0.214
24-week	1.7 (1.0, 2.3)	-	1.2 (0.4, 2.0)	-	0.5 (-0.6, 1.5)	0.378
Change from baseline to 24-week	0.4 (-1.0, 0.1)	0.102	-0.6 (-1.5, 0.2)	0.403	0.1 (-1.1, 0.8)	0.755
**Tiredness**						
Per-protocol analysis	(n=24)		(n=16)			
Baseline	3.0 (2.4, 3.7)	-	3.0 (2.2, 3.8)	-	0.0 (-1.0, 1.1)	0.938
16 weeks	2.5 (1.9, 3.1)	-	2.5 (1.7, 3.3)	-	0.0 (-1.0, 1.0)	1.000
24-week	2.4 (1.7, 3.0)	-	2.0 (1.2, 2.8)	-	0.4 (-0.6, 1.4)	0.428
Change from baseline to 24-week	-0.6 (-1.3, -0.0)	0.043	-1.0 (-1.9, -0.2)	0.020	0.4 (-1.4, 0.7)	0.482
**Sleep problems**						
Per-protocol analysis	(n=24)		(n=16)			
Baseline	1.6 (0.9, 2.3)	-	2.3 (1.5, 3.2)	-	-0.7 (-1.8, 0.4)	0.212
16 weeks	1.6 (0.9, 2.3)	-	2.0 (1.2, 2.8)	-	-0.4 (-1.5, 0.7)	0.490
24-week	1.7 (1.0, 2.4)	-	1.8 (0.9, 2.6)	-	-0.1 (-1.1, 1.0)	0.936
Change from baseline to 24-week	0.1 (-0.3, 0.4)	0.627	-0.5 (-1.1, 0.0)	0.051	0.6 (-1.3, -0.0)	0.038
**Sensitivity to cold**						
Per-protocol analysis	(n=24)		(n=16)			
Baseline	0.5 (0.1, 1.0)	-	0.9 (0.3, 1.5)	-	-0.3 (-1.1, 0.4)	0.390
16 weeks	0.4 (-0.0, 0.8)	-	0.4 (-0.1, 0.9)	-	0.0 (-0.7, 0.6)	0.847
24-week	0.5 (0.0, 0.9)	-	0.5 (0.0, 1.0)	-	0.0 (-0.7, 0.6)	0.860
Change from baseline to 24-week	0.0 (-0.4, 0.2)	0.604	-0.4 (-1.2, 0.4)	0.364	0.4 (-1.0, 0.5)	0.454
**Increased thirst**						
Per-protocol analysis	(n=24)		(n=16)			
Baseline	1.2 (0.6, 1.7)	-	1.2 (0.5, 1.8)	-	0.0 (-0.9, 0.8)	0.960
16 weeks	0.9 (0.3, 1.4)	-	0.6 (-0.1, 1.2)	-	0.3 (-0.6, 1.2)	0.475

24-week	0.8 (0.4, 1.2)	-	0.6 (0.1, 1.1)	-	0.2 (-0.4, 0.9)	0.451
Change from baseline to 24-week	-0.4 (-0.8, 0.2)	0.188	-0.6 (-1.2, 0.0)	0.056	0.2 (-1.0, 0.5)	0.484
**Increased irritability**						
Per-protocol analysis	(n=24)		(n=16)			
Baseline	1.4 (0.8, 2.0)	-	1.4 (0.7, 2.2)	-	0.0 (-1.0, 0.9)	0.965
16 weeks	1.0 (0.5, 1.5)	-	1.4 (0.8, 2.1)	-	-0.4 (-1.3, 0.4)	0.287
24-week	1.2 (0.5, 1.9)	-	0.9 (0.0, 1.7)	-	0.3 (-0.7, 1.4)	0.523
Change from baseline to 24-week	-0.2 (-0.8, 0.4)	0.468	-0.5 (-1.2, 0.1)	0.081	0.4 (-1.2, 0.5)	0.406
**Back pain**						
Per-protocol analysis	(n=24)		(n=16)			
Baseline	1.7 (1.0, 2.5)	-	2.1 (1.2, 3.0)	-	-0.4 (-1.6, 0.8)	0.480
16 weeks	1.6 (0.8, 2.4)	-	1.9 (0.9, 2.9)	-	-0.3 (-1.6, 1.0)	0.647
24-week	1.0 (0.5, 1.6)	-	1.3 (0.6, 2.0)	-	-0.3 (-1.2, 0.6)	0.570
Change from baseline to 24-week	-0.7 (-1.1, -0.2)	0.008	-0.8 (-1.9, 0.1)	0.086	0.2 (-1.1, 0.8)	0.735
**Frequent urination**						
Per-protocol analysis	(n=24)		(n=16)			
Baseline	1.6 (0.9, 2.3)	-	1.3 (0.5, 2.2)	-	0.3 (-0.8, 1.4)	0.620
16 weeks	1.4 (0.8, 2.0)	-	1.2 (0.5, 1.9)	-	0.2 (-0.7, 1.2)	0.624
24-week	1.2 (0.6, 1.8)	-	0.9 (0.1, 1.6)	-	0.3 (-0.6, 1.3)	0.483
Change from baseline to 24-week	-0.4 (-0.9, 0.1)	0.119	-0.4 (-1.4, 0.5)	0.362	0.0 (-1.0, 0.9)	0.881
**Pain in the joints**						
Per-protocol analysis	(n=24)		(n=16)			
Baseline	2.6 (1.8, 3.4)	-	2.2 (1.2, 3.2)	-	0.4 (-0.9, 1.7)	0.499
16 weeks	2.5 (1.7, 3.2)	-	1.7 (0.8, 2.6)	-	0.8 (-0.4, 1.9)	0.189
24-week	2.5 (1.6, 3.3)	-	1.6 (0.5, 2.7)	-	0.8 (-0.6, 2.3)	0.231
Change from baseline to 24-week	-0.1 (-0.8, 0.5)	0.590	-0.6 (-1.8, 0.6)	0.310	0.5 (-1.6, 0.8)	0.493
**Water retention**						
Per-protocol analysis						
Baseline	1.9 (1.3, 2.5)	-	1.0 (0.2, 1.8)	-	0.9 (-0.1, 1.9)	0.063
16 weeks	1.7 (1.0, 0.3)	-	1.1 (0.3, 1.9)	-	0.5 (-0.5, 1.6)	0.305
24-week	1.7 (1.0, 2.3)	-	0.9 (0.1, 1.7)	-	0.8 (-0.2, 1.8)	0.120
Change from baseline to 24-week	-0.2 (-0.8, 0.3)	0.341	-0.1 (-0.8, 0.5)	0.682	0.1 (-0.7, 0.9)	0.770
**Foot problems**						
Per-protocol analysis	(n=24)		(n=16)			
Baseline	1.5 (0.9, 2.1)	-	1.1 (0.3, 1.8)	-	0.4 (-0.6, 1.4)	0.381
16 weeks	1.1 (0.5, 1.8)	-	1.3 (0.5, 1.8)	-	-0.2 (-1.2, 0.9)	0.722
24-week	1.5 (0.9, 2.2)	-	1.2 (0.4, 2.0)	-	0.3 (-0.7, 1.4)	0.522
Change from baseline to 24-week	0.0 (-0.4, 0.5)	0.857	0.1 (-0.9, 1.2)	0.752	-0.1 (-0.9, 1.1)	0.824
**Sensitivity to heat**						
Per-protocol analysis	(n=24)		(n=16)			
Baseline	1.5 (0.7, 2.4)	-	1.7 (0.6, 2.7)	-	-0.2 (-1.5, 1.2)	0.828
16 weeks	0.9 (0.4, 1.4)	-	0.7 (0.1, 1.3)	-	0.2 (-0.6, 1.0)	0.629
24-week	1.3 (0.5, 2.0)	-	1.0 (0.1, 1.9)	-	0.3 (-0.9, 1.4)	0.653
Change from baseline to 24-week	-0.2 (-0.9, 0.3)	0.328	-0.7 (-1.6, 0.3)	0.154	0.4 (-1.4, 0.6)	0.439
**Snoring**						
Per-protocol analysis	(n=24)		(n=16)			
Baseline	2.4 (1.5, 3.3)	-	2.1 (1.1, 3.2)	-	0.3 (-1.1, 1.6)	0.717
16 weeks	2.2 (1.5, 3.0)	-	1.6 (0.6, 2.5)	-	0.6 (-0.5, 1.8)	0.275
24-week	1.7 (1.0, 2.4)	-	1.6 (0.7, 2.5)	-	0.1 (-1.0, 1.2)	0.879
Change from baseline to 24-week	-0.7 (-1.5, 0.2)	0.126	-0.5 (-1.5, 0.4)	0.253	0.2 (-1.1, 1.4)	0.794
**Increased appetite**						
Per-protocol analysis	(n=24)		(n=16)			
Baseline	1.9 (1.3, 2.6)	-	1.5 (0.7, 2.3)	-	0.4 (-0.6, 1.5)	0.426
16 weeks	1.8 (1.2, 2.4)	-	1.6 (0.8, 2.3)	-	0.2 (-0.7, 1.2)	0.560
24-week	2.1 (1.5, 2.7)	-	1.0 (0.3, 1.8)	-	1.1 (0.1, 2.1)	0.027

Change from baseline to 24-week	0.2 (-0.4, 0.8)	0.504	-0.5 (-1.3, 0.3)	0.223	0.7 (-1.7, 0.3)	0.172
**Leakage of urine**						
Per-protocol analysis	(n=24)		(n=16)			
Baseline	0.3 (0.0, 0.7)	-	0.3 (-0.2, 0.7)	-	0.0 (-0.4, 0.6)	0.749
16 weeks	0.3 (0.0, 0.6)	-	0.3 (-0.0, 0.7)	-	0.0 (-0.5, 0.4)	0.922
24-week	0.3 (-0.1, 0.6)	-	0.5 (0.1, 0.9)	-	-0.3 (-0.7, 0.2)	0.275
Change from baseline to 24-week	0.0 (--0.3, 0.1)	0.328	0.2 (-0.2, 0.8)	0.283	-0.3 (-0.1, 0.8)	0.118
**Lightheadness**						
Per-protocol analysis	(n=24)		(n=16)			
Baseline	0.6 (0.1, 1.2)	-	1.1 (0.1, 0.9)	-	-0.4 (-1.3, 0.4)	0.305
16 weeks	0.5 (0.1, 0.9)	-	0.6 (0.1, 1.0)	-	-0.6 (-0.7, 0.6)	0.839
24-week	0.7 (0.2, 1.2)	-	0.6 (-0.0, 1.1)	-	0.2 (-0.6, 0.9)	0.667
Change from baseline to 24-week	0.1 (-0.2, 0.4)	0.575	-0.5 (-1.1, 0.1)	0.081	0.6 (-1.2, -0.0)	0.040
**Increased sweating**						
Per-protocol analysis	(n=24)		(n=16)			
Baseline	1.3 (0.5, 2.0)	-	1.7 (0.8, 2.6)	-	-0.4 (-1.6, 0.8)	0.502
16 weeks	0.8 (0.4, 1.3)	-	0.6 (0.1, 1.2)	-	0.2 (-0.5, 0.9)	0.557
24-week	1.0 (0.5, 1.6)	-	1.2 (0.5, 1.9)	-	-0.2 (-1.0, 0.7)	0.728
Change from baseline to 24-week	-0.3 (-0.9, 0.6)	0.398	-0.5 (1.1, 0.1)	0.116	0.2 (-1.1, 0.6)	0.575
**Loss of sexual desire**						
Per-protocol analysis	(n=24)		(n=16)			
Baseline	1.7 (0.9, 2.4)	-	1.7 (0.8, 2.6)	-	0.0 (-1.2, 1.1)	0.971
16 weeks	1.2 (0.8, 1.7)	-	0.6 (0.0, 1.1)	-	0.6 (-0.1, 1.4)	0.077
24-week	1.4 (0.8, 1.9)	-	0.8 (0.1, 1.5)	-	0.5 (-0.3, 1.4)	0.217
Change from baseline to 24-week	-0.3 (-0.8, 0.2)	0.245	-0.9 (-1.6, -0.1)	0.029	0.6 (-1.4, 0.3)	0.184
**Decreased physical stamina**						
Per-protocol analysis	(n=24)		(n=16)			
Baseline	2.6 (1.9, 3.3)	-	2.4 (1.5, 3.3)	-	0.2 (-0.9, 1.4)	0.716
16 weeks	1.5 (1.0, 1.2)	-	0.7 (0.1, 1.3)	-	0.8 (0.0, 1.6)	0.044
24-week	1.4 (0.8, 2.0)	-	0.8 (0.0, 1.5)	-	0.6 (-0.4, 1.5)	0.211
Change from baseline to 24-week	-1.2 (-2.0, -0.4)	0.005	-1.6 (-2.6, -0.6)	0.003	0.4 (-1.6, 0.8)	0.525
**Skin irritation**						
Per-protocol analysis	(n=24)		(n=16)			
Baseline	1.3 (0.6, 1.9)	-	0.7 (-0.1, 1.5)	-	0.6 (-0.4, 1.6)	0.242
16 weeks	0.7 (0.2, 1.2)	-	0.6 (-0.0, 1.3)	-	0.1 (-0.8, 0.9)	0.844
24-week	1.1 (0.6, 1.6)	-	0.2 (-0.4, 0.9)	-	0.9 (0.0, 1.7)	0.040
Change from baseline to 24-week	-0.2 (-0.9, 0.5)	0.519	-0.5 (-1.1, 0.1)	0.114	0.3 (-1.2, 0.7)	0.553
